# Do gluten peptides stimulate weight gain in humans?

**DOI:** 10.1111/nbu.12558

**Published:** 2022-05-30

**Authors:** Fred Brouns, Peter R. Shewry

**Affiliations:** ^1^ School for Nutrition and Translational Research in Metabolism Maastricht University Maastricht The Netherlands; ^2^ Rothamsted Research UK

**Keywords:** bioactive peptides, exorphins, gluten, weight gain

## Abstract

Observations from animal and in vitro laboratory research, and anecdotal evidence, have led to the suggestion that gluten consumption stimulates weight gain by the presence of peptides expressing opioid activity. Another proposed mechanism is that gluten peptides decrease resting energy expenditure resulting in a positive energy balance. In order to induce such effects in vivo, intact food peptides must be absorbed in sufficient quantities, remain intact in the blood for sufficient time to have long‐lasting biological activity and bind to receptors involved in appetite, satiety and energy regulation. However, although peptides from food may pass from the intestine into the blood in extremely low quantities, they are generally rapidly degraded by plasma and vasculum‐bound aminopeptidases, resulting in very short half‐lives and loss of bioactivity. At present, gluten peptide sequences that influence regulators of energy metabolism have not been identified. Furthermore, data on the quantitative absorption of gluten peptides in the blood stream, their stability and lasting bioactivity are also lacking. Therefore, there is no evidence for proposed effects on driving appetite by the brain, nor on energy expenditure and weight gain. Furthermore, the level of overweight observed in various countries appears to be independent of the level of wheat consumption, and abundant observational evidence in humans shows that the levels of gluten consumption are neither related to daily calorie intake nor to BMI. This narrative review therefore discusses the proposed effects of gluten on bodyweight (BW) and putative biological mechanisms in the light of the current evidence.

## BACKGROUND

**FIGURE 1:**
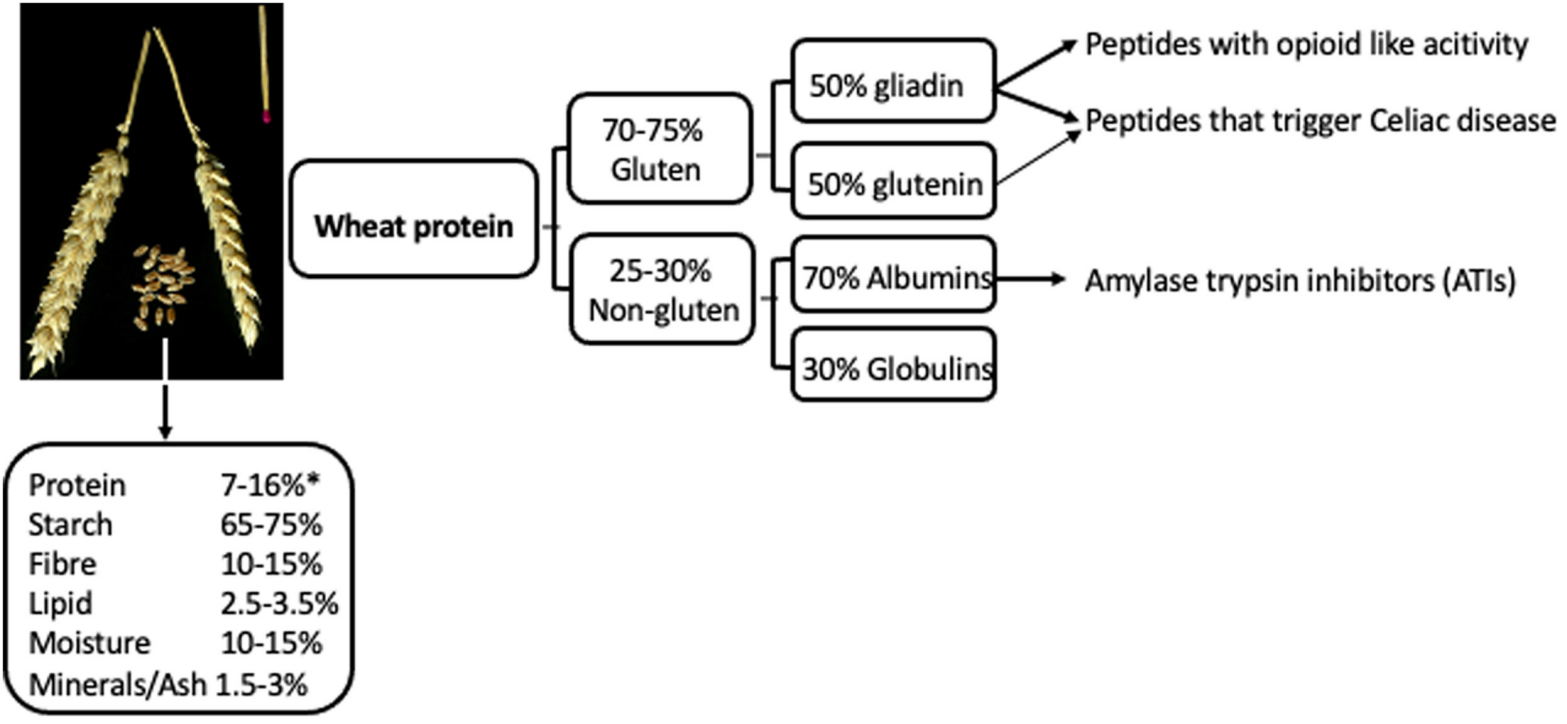
Composition of the wheat kernel and of wheat protein, based on data from (Kucek et al., 2015; Zorb et al., 2018)

Wheat, rice and maize are the three major cereals used as staple foods around the globe for both human and livestock nutrition. At present, about 40% of total cereal production is used in the production of foods for human use and 35% is used in livestock feed (Poutanen et al., 2021). Cereals are rich in starch and a good source of protein, which makes them significant contributors to overall carbohydrate and protein intake in humans. Figure 1 gives a simplified overview of wheat nutritional composition and major protein fractions, showing the source of gluten peptides and amylase trypsin inhibitors (ATIs), which may both trigger adverse reactions in susceptible individuals. ATIs are proteins that are relatively resistant to digestion. Their occurrence, function and health effects are extensively reviewed elsewhere (Geisslitz et al., 2022).

About 95% of the wheat grown worldwide is *Triticum aestivum* var. *aestivum*, often referred to as ‘bread wheat’, which is mainly used for bread and other bakery products. Most of the remaining 5% is durum wheat (*Triticum turgidum* var *durum*) used for pasta making. Other types of wheat such as spelt (*Triticum aestivum* var. *spelta*), emmer (*Triticum turgidum* var.*dicoccum*) and einkorn (*T. monococcum* var. *monococcum*), are only grown in very small amounts. Variation in the protein content of cereals results from genetic differences (species and cultivars), levels of nitrogen fertiliser used, environmental growing conditions (Godfrey et al., 2010; Kasarda, 2013) and analytical methods (Schalk et al., 2017). The values in composition tables used by nutritionists are generally within the following ranges: hard wheat (for breadmaking) 11%–16%, soft wheat (for cakes, biscuits and pastry) 7%–11% (Kasarda, 2013), spelt 11%–15%, durum 10%–14%, emmer 10%–13%, rye 11%–16%, barley 8%–18% and oat 16%–19%. All types of wheat contain gluten, accounting for ~70%–80% of the total proteins in bread wheat (*Triticum aestivum* var. *aestivum*), ~78% in spelt, ~77% in durum wheat and ~70% in emmer (Geisslitz et al., 2019; Schalk et al., 2017). Barley and rye contain proteins closely related to gluten (and are therefore listed as gluten‐containing grains), but they are given specific names: hordein in barley (~50%–55%) and secalin in rye (~47%–50%) (Gellrich & Schieberle, 2003; Schalk et al., 2017). The avenin proteins in oats are less closely related to gluten and hence oats are not classified as gluten‐containing. However, oats can be contaminated with gluten‐containing grains/flours in the food production, transport and processing chain.

Over recent years, popular books and social media postings have suggested that gluten consumption causes overweight and related chronic diseases (type 2 diabetes, cardiovascular disease). However, the data used to support such claims are often questionable. For example, data from a clinical study that showed that eliminating gluten consumption from the diet resulted in weight loss (in 25 coeliac patients) (Murray et al., 2004) was used as evidence that gluten ingestion resulted in weight gain (Davis, 2012). However, the study that was referred to (Murray et al., 2004) also noted that 91 patients gained weight after gluten exclusion, which was ignored. Miller Jones (Miller‐Jones, 2012) has challenged many such claims and many questions remain unanswered.

In their report entitled *Sustainable healthy diets: guiding principles*, the Food and Agriculture Organization of the United Nations and World Health Organization (2019) conclude as follows: ‘Studies of food and health relationships have consistently highlighted associations between low intakes of plant‐based foods and high intake of animal products and ultra‐processed foods, and poor health outcomes. These findings point to plant‐ versus animal‐based diets and degree of food processing as priority characteristics for analysing dietary patterns in the context of sustainability considerations’. The report emphasises the importance of increasing the intake of plant foods, including whole grains, a recommendation that has been implemented in most food‐based dietary guidelines worldwide (Martini et al., 2021). In this light, it is important that public concerns that grains containing gluten induce weight gain and related chronic diseases are addressed thoroughly.

If gluten is a causative factor for weight gain, what are the underlying mechanisms and what do well‐controlled intervention studies tell us? What do observational studies tell us? Is there a relation between the amounts of gluten‐containing foods that are consumed and bodyweight (BW)? We will address these questions below.

## DOES GLUTEN CONSUMPTION STIMULATE ‘APPETITE FOR MORE’?

### Is there a plausible mechanism?

The mechanism by which gluten is suggested to cause increased consumption is that the digestive break down of gluten in the small intestine leads to the formation of peptides, some of which have structural similarities with endogenous secreted opioid peptides (endorphins) (Meisel, 1997; Teschemacher et al., 1997), (Table 1). Two types of endogenous opioid peptides exist, one containing Try‐Gly‐Gly‐Phe as the message domain (enkephalins, endorphins, dynorphins) and the other containing the Tyr‐Pro‐Phe/Trp sequence (endomorphin‐1 and ‐2) (Zadina, 2002). When the peptides come from an external source, for example, from food, they are called exorphins (an alternative name sometimes used is ‘nutropoids’). When derived from gluten, the name ‘gluten exorphins’ is used. Note that all of these peptides have a chain length of four or more amino acids, which is important with respect to strong limitations in bioavailability (see further below). Early work reported opioid activity in vitro, using peptide fractions of a wheat gluten hydrolysate (Huebner et al., 1984). Depending on the receptor, the binding of opioid food peptides can lead to agonistic (stimulating) and antagonistic (inhibiting) effects on appetite, satiety and related food intake (Gosnell & Levine, 2009). For example, casomorphins (derived from milk casein) and gluten exorphins are classified as opioid agonists, whereas most other identified food exorphins are classified as opioid antagonists (Duraffourd et al., 2012; Teschemacher, 2003). Depending on the type of opioid receptor involved (there is a wide range of receptors [Bodnar, 2021]), effects may differ as shown in mice experiments where δ and μ opioid peptides derived from food proteins appeared to have opposite effects on the quantitative intake of normal and high‐fat diets (Yoshikawa, 2015). The protein sources, which have been most studied in this respect are milk casein, soy protein, rice albumin and wheat gluten (Fukudome et al., 1997; Fukudome & Yoshikawa, 1992; Fukudome & Yoshikawa, 1993; Pica et al., 2021; Teschemacher, 2003).

**TABLE 1:** Opioid peptides: Two endorphin examples and some peptides resulting from enzymatic in vitro digestion of gluten and soybean (Fukudome & Yoshikawa, 1992; Fukudome & Yoshikawa, 1993; Kaneko et al., 2010)

Endorphin	Amino acid sequence of receptor message domain
Endomorphin‐1	Tyr‐Pro‐Trp‐Phe
Endomorphin‐2	Tyr‐Pro‐Phe‐Phe
Gluten Exorphin (GE) Peptide	
GE‐A4	Gly‐Tyr‐Tyr‐Pro
GE‐B4	Tyr‐Gly‐Gly‐Trp
GE‐A5	Gly‐Tyr‐Tyr‐Pro‐Thr
GE‐B5	Tyr‐Gly‐Gly‐Trp‐Leu
GE‐C	Tyr‐Pro‐Ile‐Ser‐Leu
Soybean morphin‐5	Tyr‐Pro‐Phe‐Val‐Val

The suggestion that wheat gluten causes increased food intake goes back to early observations that the secretion of opioids in the human brain is associated with feelings of pleasure or euphoria. Similarly, consuming a very nice meal, which is associated with feelings of pleasure, has been suggested to drive consumption by the action of endorphins (’I like to eat more of this') (Morley & Levine, 1982). Following on from this, it is easy to assume that opioid peptides resulting from gluten digestion and absorbed into the blood will reach the brain and lead to hedonic feelings and increased appetite. However, this fails to consider the complexity of hunger, satiety and appetite regulation.

The presence of opioid peptides in (fermented) food, in protein hydrolysates and/or in the gastrointestinal tract during digestion does not necessarily mean that these are absorbed in sufficient amounts, remain stable after absorption and reach the brain to elicit a biological effect. Early studies (Gardner, 1982), in which the absorption of peptides present in protein hydrolysates was studied during luminal perfusion of isolated rat small intestine, concluded that ‘while the passage of intact peptides across the small intestine probably occurs on too small a scale to be of major nutritional relevance, some passage does appear to occur during assimilation of at least some proteins, which requires further study’. Thirty‐two years later, Miner‐Williams et al. (2014) concluded that there is little unequivocal evidence that bioactive peptides longer than three amino acids can cross the gut wall and enter the hepatic portal vein and systemic circulation in sufficient amounts to cause biological effects in vivo. Fernandez‐Tome and Hernandez‐Ledesma (2020) concluded that peptides with bioactivity in vitro do not always correlate with in vivo activity and that effects largely depend on their ability to remain intact after digestive processes. Accordingly, the identification of the gluten exorphins C5 and A5 in in vitro gastric or intestinal digestates of spaghetti (Takács et al., 2018) does not prove that they induce biological effects. Below we will discuss this matter step by step.

### Actions of opioid peptides in the human body

Experiencing happiness is associated with the release of endorphins in the brain. However, opioids have multiple biological effects. Endogenous opioids are well known to reduce the gastrointestinal digestion, transit and absorption of meals, due to the reduction of gastrointestinal motility (De Luca & Coupar, 1996). It is suggested that this is due to the action of opioid receptors present in the myenteric and the submucosal plexus of the intestinal wall, and in the portal vein (Sobczak et al., 2014). Similarly, endorphins are known to reduce pain perceptions and morphine medication to counteract pain is known to be associated with reduced intestinal motility, reduction of gastric emptying and feelings of fullness and nausea. In contrast to stimulating appetite, these responses would be expected to result in loss of appetite and reduced food intake (Sobczak et al., 2014).

## DOES FOOD PROTEIN DIGESTION INDUCE APPETITE AND MORE FOOD CONSUMPTION?

A minimal protein intake of 0.8 g/kg BW/day is important to maintain nitrogen balance and optimal organ and muscle function. Although the quality scores (Digestible Indispensable Amino Acid Score [DIAAS]) of single plant proteins, including cereals, may be lower due to essential amino acids that are less abundant than in animal sources, it should be noted that humans consume proteins from various sources, which will compensate for limiting amino acids, for example, legume proteins will compensate for the deficiency of lysine in cereal proteins (Adhikari et al., 2022; Bailey & Stein, 2019; Millward et al., 2002). For example, a breakfast meal comprised of 40% cereals and 60% milk, will result in a calculated DIAAS of 107, resulting in excellent overall protein quality (Rutherfurd et al., 2015). But, do proteins stimulate eating? Based on the assumption that protein digestion will always lead to increased exposure to opioid‐like peptides, it could be suggested that cereal protein digestion will always result in increased appetite. This appears not to be the case. There is abundant evidence from human intervention studies that high protein diets and diets enriched with protein hydrolysates (primarily composed of peptides) may reduce appetite, gastric emptying and food intake (Drummen et al., 2018; Duraffourd et al., 2012; Janssen et al., 2011). The endogenously secreted satiety regulating peptide hormones, such as GLP‐1 and PYY are released from the small intestine during protein digestion and absorption but are subject to very rapid clearance (2–4 min, Table 2, (Belza et al., 2013). It has been suggested that these hormones act synergistically with peptides derived from food digestion to result in greater satiation than with carbohydrate and fat alone (Astrup et al., 2015; Bensaid et al., 2003; Hansen et al., 2021; Kim et al., 2016). The composition of the overall diet, which always contains a mixture of proteins, carbohydrates and lipids, and its interaction with factors that regulate metabolism, ultimately determine the functional and health outcomes (Drummen et al., 2018). In particular, it should be noted that endorphins have very rapid clearance and loss of bioactivity. Accordingly, continuous endogenous secretion is required to maintain elevated levels for a lasting effect. Similarly, continuous long‐term absorption of bioactive peptides from food would be required to maintain circulating levels, which appears not to be the case.

**TABLE 2:** Endogenously produced polypeptide hormones with regulatory function show a very rapid clearance (*t*_1/2_) from blood

Polypeptide hormones	Secreted by	Amino acids	*t*_1/2,_ min
Insulin	Pancreas	51	4–6
Glucagon	Pancreas	29	3–5
Glp‐1	Ileum L cells	30	1–3
GIP	Duodenum, K cells	42	2–5
PYY	Ileum, L cells	36	3–4
CCK	Duodenum, L cells	8–58	2–3
VIP	Intestinal immune cells and neurons	28	1–2

*Note*: Abbreviations: GLP1, glucagon‐like peptide 1; GIP, gastric inhibitory polypeptide; PYY, peptide‐YY; CCK, cholecystokinine; VIP, vasoactive intestinal peptide.

## CAN FOOD EXORPHINS STIMULATE APPETITE THROUGH EFFECTS ON THE BRAIN?

There are four critical questions in this respect. These are listed below:

### Can food exorphins present in blood induce biological effects?

By supplying chemically synthesised gluten exorphins (GE‐B5, see Table 1) by intravenous infusion in rats, it was shown that food exorphins in blood can exert biological effects at different dosages (Fanciulli et al., 2003). The highest dose of 3 mg/kg BW (but not lower doses) was shown to influence the secretion of the hormone prolactin. Takahashi et al. (2000) administered gluten exorphin‐5 directly into the brain (intraventricular) of mice and observed mild pain‐reducing effects, but again only with the highest dose. It was suggested that the absence of effects at lower dosages may be due to the rapid degradation and loss of bioactivity of the peptides.

### Are gut luminal exorphins absorbed to a significant extent?

**FIGURE 2:**
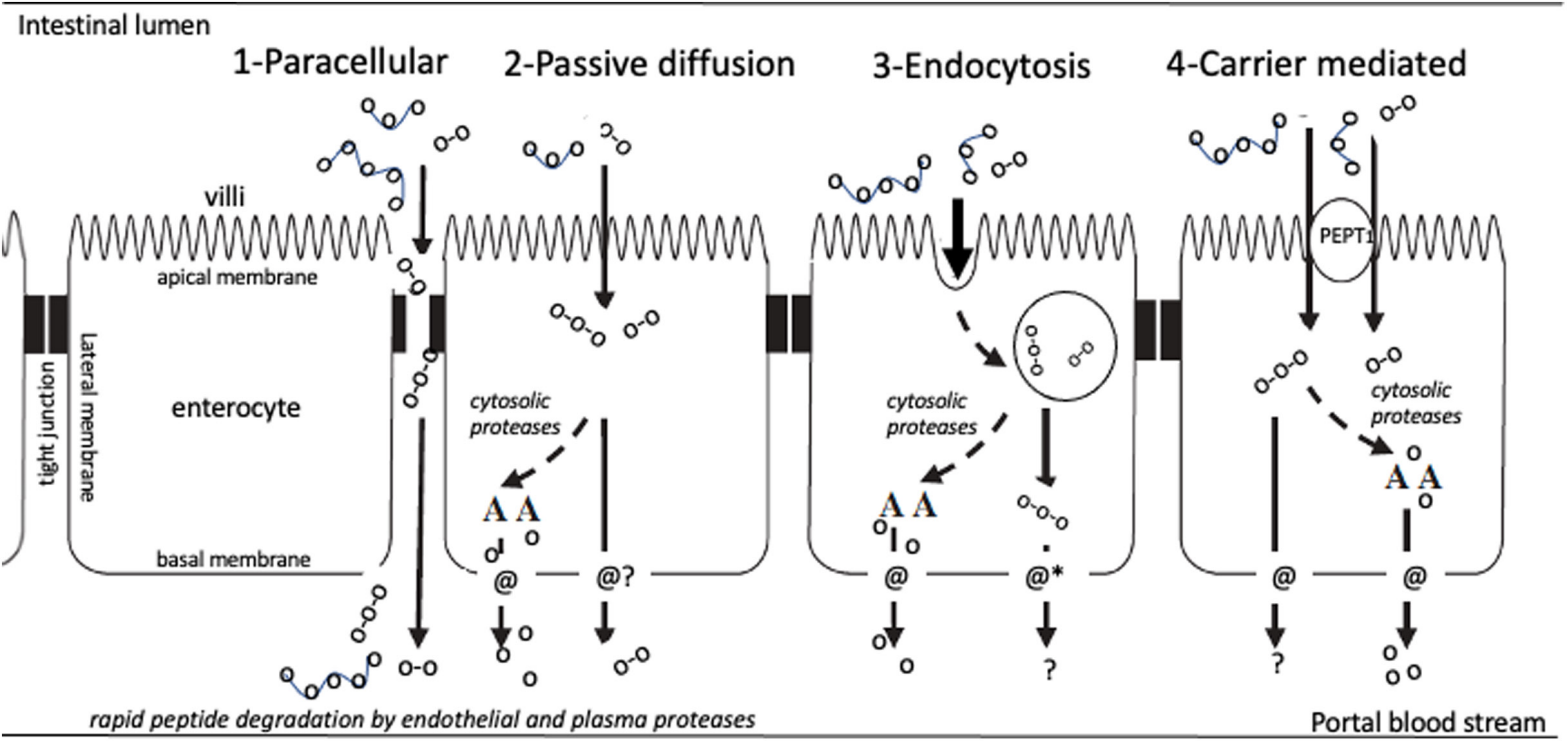
Potential pathways of amino acid and peptide absorption in the small intestine. (1) Paracellular through widened tight junctions, (2) passive diffusion through the enterocyte, (3) transcellular endocytosis, followed by carrier transport or suggested peptide cargo*‐permeability and (4) transcellular transport by carrier‐mediated passage (*It is unlikely that peptides will passively diffuse across the cell membrane, but altering their physical properties (such as conformational flexibility and polarity) has been proposed to improve their permeability, also referred to as peptide cargo (Yang & Hinner, 2015) figure modified from (Miner‐Williams et al., 2014))

Food exorphins will only exert opioid effects that impact on appetite, satiety and food intake when they are present in sufficient quantities and for sufficient time in blood. Possible pathways for the absorption of peptides from the gut are depicted in Figure 2. A possible route is increased permeability, due to the widening of the tight junctions between the cells of a single layer of enterocytes that represent the intestinal epithelial surface. Under normal circumstances, these tight junctions are extremely narrow to allow the passage of water and small molecules such as electrolytes and water‐soluble small peptides. The tight junction space is regulated by a network of synergistically acting transmembrane proteins (Sturgeon & Fasano, 2016) and can be increased due to various factors that affect this network, such as the use of nonsteroidal anti‐inflammatory drugs, alcohol, intense physical (lasting and dehydrating) exercise, anxiety stress, bacterial overgrowth and a range of chronic inflammatory diseases that affect intestinal integrity (Camilleri, 2019; Elamin et al., 2014; Fasano, 2020). These factors and related disease pathologies are known to induce the release of zonulin, a (47 kDa) protein secreted by enterocytes, which in concert with other factors reversibly modulates tight junction permeability by increasing its space. In addition to the factors mentioned above, luminal gliadin exposure has also been shown to induce zonulin release and increase permeability and is proposed (among other factors) to be involved in the aetiology of coeliac disease (CD) (Fasano, 2020; Sturgeon & Fasano, 2016).

CD only develops in a small proportion of individuals who consume gluten (at present ~1% of the global population) and it has been noted that increased gut permeability could not be detected in individuals self‐reporting gluten sensitivity, which has related gastrointestinal symptoms (Biesiekierski et al., 2011). The latter shows that despite its potential to increase zonulin, gluten exposure does not compromise the gut barrier in most individuals. That gluten exorphins can cross a disordered intestinal epithelium into blood was shown by Pennington et al. (2007), after consumption of wheat protein by newly diagnosed CD patients who were not yet on a gluten‐free diet. To put this observation in a correct perspective it should be emphasised that untreated CD patients are known to have an inflamed, damaged intestinal barrier function, allowing macromolecules (including intact proteins, peptides and bacteria) to pass paracellularly (i.e. between cells) from the intestinal lumen into blood (Castell et al., 1997; Heyman, 2001; Heyman, 2005; Uhde et al., 2016). However, the presence of larger peptides in blood under such specific pathophysiological circumstances does not show that gluten exorphins are absorbed under normal physiological conditions, in which the paracellular route is minimal and transport takes place primarily transcellularly, during which the peptides are exposed to cytosolic peptidases that will result in their degradation.

Ozorio et al. (2020) reported ex vivo studies of piglet small intestinal (jejunum) tissue in the Ussing chamber model. An Ussing chamber is composed of two compartments separated by a tiny sheet of gut mucosa obtained from either biopsy or tissue culture. One compartment represents the apical (intestinal lumen) side, the other the basolateral side of the mucosa (see also Figure 2). The passage of ions, amino acids, peptides, etc, across the epithelium, can be measured by disappearance from the apical side and appearance on the basolateral side. They observed that only very small amounts (0.6%–3.35%) of peptides originating from the digestion of lactoglobulin and casein digestion could pass through the epithelial barrier and reach the basolateral compartment, which in vitro represents the blood space (peptides shorter than six amino acids could not be detected due to methodological limitations). This is in agreement with other studies using Ussing chambers, which showed only low absorption and in vivo studies that show a very low entry of di‐ and tripeptides into hepatic portal vein in experimental animals as reviewed by Miner‐Williams et al. (2014).

Stuknytė et al. (2015) observed the presence of two gluten exorphins after in vitro digestion of pasta and bread, respectively. These peptides, called GE‐A5 and GE‐C5 (for peptide sequences see Table 1), were present in the ranges 0.747–2.192 mg/kg and 3.201–6.689 mg/kg, respectively. In further studies, they exposed a monolayer culture of human Caco‐2 cells to synthetic GE‐A5 and GE‐C5 to determine in vitro absorption. They observed that the peptides were extensively degraded by the Caco‐2/HT‐29 co‐culture peptidases and that only 3% and 1% of GE‐A5 and GE‐C5, respectively, were ‘absorbed’ after 120 min of incubation. It should be noted that the in vitro cell culture models, which are used to predict absorption in vivo have a number of potential limitations, including the absence of passage through the stomach (with exposure to gastric acid) and of a mucus layer and lower activities of enzymes associated with the brush border (Bolte et al., 1998; Foltz et al., 2010). This is considered to be one of the reasons that the observed passage of components into portal blood in vivo is much lower than in controlled in vitro studies. Casein‐derived opioid peptides (b‐casomorphins, chain length *n* = 5–11) generally did not survive intestinal and plasma degradation, as shown in rabbits (Mahe et al., 1989; Petrilli et al., 1984) and consequently are only detectible in extremely low amounts in the blood, as reviewed by Vermeirssen et al. (2004). More recently, using a multiple cannulated pig model, which allowed for continuous sampling of portal blood, the postabsorption concentration of (angiotensin‐converting enzyme) ACE‐inhibiting peptides (chain length 3) was very low: in pico‐nanomolar concentration corresponding to about 0.1% of the ingested dose and a thousandfold (Miner‐Williams et al., 2014) below the concentration required to induce a biological effect (Foltz et al., 2010; van der Pijl et al., 2008). As a consequence, their presence in in vitro digested food was not considered to be relevant for the situation in humans, which requires data on in vivo human absorption and biological activity (Foltz et al., 2010).

### Do absorbed exorphins remain stable over time?

The opioid peptides, which are released during the digestion of various food proteins, are relatively small (chain length of 3–7 amino acids [Bakalkin et al., 1992]). The known gluten exorphins have chain lengths of four and five amino acids, respectively (Fukudome & Yoshikawa, 1992; Fukudome & Yoshikawa, 1993) (Table 1). Most peptides are broken down completely by the combined action of gastric acid hydrolysis and gastric and pancreatic proteases. Di‐ and tripeptides that escape this luminal digestion may pass into the enterocytes but will generally be degraded subsequently intracellularly by cytosolic peptidases. Any peptide that still passes into the blood will undergo rapid and complete degradation by vascular endothelial tissue peptidases and plasma peptidases, resulting in very short half‐life times in blood, which in the case of many peptides are only a few minutes. A similar short half‐life was observed for opioid peptides (Gambus et al., 1998; Mosnaim et al., 2000; Stuknytė et al., 2015). Consequently, no lasting biological impact can be expected (critically reviewed by Miner‐Williams et al. (2014)).

Takahashi et al. (2000) observed effects of gluten exorphin‐C5 on pain reduction when administered directly into the brain of mice (at 30 and 300 μg/kg, respectively) but no such effects when administered orally in doses of 30 and 300 mg/kg BW, which would correspond to oral doses in humans of 2.44 and 24.4 mg/kg, respectively, or 170 mg and 1.7 g for a 70 kg human, which is unrealistically high (Naïr & Jacob, 2016). Small effects were observed on learning and stress parameters but only at the highest oral dose. No measurements were made of blood levels after oral administration of GE‐C5 and the paper is subject to questions regarding methodology, statistics and interpretation. Considering the data cited above, gluten exorphins with chain lengths of four and five amino acids are not expected to be absorbed intact in humans in a sufficient quantity and even if absorbed, to remain stable enough to induce a measurable biological effect resulting in a quantitative change in food consumption.

### What are the effects of the presence of peptides in the intestinal lumen or enterocyte?

Could exorphins have effects on humans by direct effects on local cells and neuronal networks within the intestine, even if they are not present in blood? This question is relevant because angiotensin‐converting enzyme inhibiting peptides have been shown to result in blood pressure lowering, despite their virtual absence from blood post ingestion (van der Pijl et al., 2008). It has been postulated that this effect may be caused by effects on opioid receptors present in the intestinal wall, which affect fluid fluxes between the blood and gut and thereby on blood pressure (extensively discussed by Miner‐Williams et al. (2014).

It has been shown that soybean morphine supplied to mice suppresses intestinal transit and reduces food intake through the action of the mu‐opioid receptor associated with the intestinal wall (Kaneko et al., 2010). Similarly, the supply of wheat gluten hydrolysate was shown to affect the intestinal secreted satiety hormone peptide‐YY and to suppress food intake in rats (Chen et al., 2018). Lang et al. (1998) compared the effects of egg albumin, casein, gelatin, soy protein, pea protein and wheat gluten on the induction of satiety in humans. The test meal contained 74.4 g of gluten (for comparison, the mean gluten intake in western countries is 5–20 g [Biesiekierski, 2017]). The results showed that all meals induced short‐term satiety, which, however, did not have effects on the 24‐h energy intake or on postprandial plasma glucose and insulin concentrations. Gluten did not differ from the other protein sources studied. Of particular interest is an early human intervention study (Morley et al., 1983) in which the effects of gluten, hydrolyzed gluten and hydrolyzed gluten plus the opiate blocker naloxone were studied. Exposure to hydrolyzed gluten (known to contain gluten exorphins [Teschemacher, 2003]) prolonged intestinal transit time, which was reversed by concomitant administration of naloxone, which suggests the presence of opioid peptides. No effects could be demonstrated on serum gastrin, cortisol, carbohydrate metabolism, or small bowel mucosal integrity. No effects of ‘exorphins present in the gluten hydrolysate’ on the quantity of calories ingested or on the perception of satiety could be demonstrated. From these observations, it appears that the intestinal presence of gluten exorphins does not result in stimulation of appetite through local stimuli. Thus, the suggestion that wheat protein, of which about 70%–75% is gluten proteins, stimulates appetite and causes increased food intake is not supported by the evidence.

### Does the act of eating stimulate endorphin release?

Recently, the hypothesis that food endorphins drive the consumption of more food due to the experience of pleasure has been challenged (Tuulari et al., 2017). It was shown that eating a delicious pizza was significantly associated with experiencing pleasant feelings and that the ingestion of a tasteless energy‐matched drink was not. Despite the difference in liking (hedonic responses), the nonpalatable drink induced a higher release of endogenous opioids in the brain. This observation clearly does not support the suggestion that gluten exorphins stimulate food intake because of hedonic effects.

## IS THERE ANOTHER MECHANISM BY WHICH GLUTEN MAY IMPACT ON WEIGHT GAIN?

Recent studies in mice have shown increased weight gain after gluten inclusion in the diet, compared with gluten‐free diets, an effect that was suggested to be caused by changes in metabolic rate (Freire et al., 2016; Soares et al., 2013). However, it is important to consider this observation in relation to the effects of gluten in humans. In a recent study entitled *Inconsistent effects of gluten on obesity* (Silva et al., 2020), 40 overweight women free from CD were enrolled in a single‐blind crossover diet study in which either a gluten‐free muffin (GF‐M) or a gluten‐containing muffin (GLU‐M, 24 g gluten) was consumed, each for 4 weeks. Energy intakes remained similar during both periods and no effects on body fat, fat‐free mass and resting energy expenditure due to the additional gluten intake were found. Although a subgroup of individuals (28% of the study population) carrying the Hp2‐2 genotype (representative of overexpressing zonulin) exhibited a lower resting energy expenditure (REE), no changes in BW were observed. Despite this, the authors concluded that the results indicate the importance of zonulin as a mediator of gluten's obesogenic effects. This report raises concerns since single measurements of REE (or resting metabolic rate, RMR) appear to have low predictability in terms of long‐term prediction of weight gain. According to Lam & Ravussin, (2017), the relationships between RMR and obesity are inconsistent, with RMR reported to show negative, positive or no correlations with obesity. Zonulin was not measured by Silva et al. (2020) and no mechanistic explanation was given as to how zonulin, which is involved in the regulation of tight junction permeability, may have effects on RMR in individuals with the Hp2‐2 genotype. In the publication (Silva et al., 2020), reference is made to two experimental studies, which showed gluten‐induced weight gain in mice that were fed a high‐fat diet with the aim of inducing obesity (Freire et al., 2016; Soares et al., 2013). The first study (Soares et al., 2013) compared a diet containing gluten with a gluten‐free diet, given to the mice for a period of 8 weeks. Up‐regulation of several genes related to lipid metabolism was observed, including PPARγ, which regulates enzymes that play a role in fatty acid oxidation and thermogenesis of brown adipose tissue. Soares et al. (2013) in turn referred to Luciani et al. (2010) who showed that specific gluten peptides are able to downregulate PPARγ in vitro in ‘gliadin sensitive’ Caco‐2 cells and epithelial cells of CD patients, obtained by biopsy. From this it was inferred that beneficial effects on lipid metabolism and rapid mitochondrial fatty acid oxidation should take place when abstaining from gluten, leading to less weight gain. However, these metabolic parameters were not measured. Furthermore, the up or down regulation of specific genes related to lipid metabolism does not necessarily translate into BW changes. For example, studies in mice with knock out of PPARγ, leading to complete abolishment of PPARγ activity, did show a reduction in brown adipose cell size and stored lipid droplets, when compared to wild type control animals, but did not result in a difference in BW and body composition (Lasar et al., 2018).

Silva et al. (2020) also refer to another study that claimed that gluten intake reduced energy expenditure and the thermogenic capacity of adipose tissue in obese mice fed a high‐fat diet (Freire et al., 2016). They used technetium labelled (de Barros et al., 2010; Faintuch et al., 2008) hydrolyzed wheat gluten, containing a wide variety of peptides (molecular weight distribution, 10.1% > 2000, 57.9% 500–2000, 32.0% < 500) and used scintigraphy to show the presence of the label in blood, liver and visceral adipose tissue. This was taken as evidence that gluten peptides were absorbed and caused the effects on REE.

However, none of these studies made quantitative measurements of peptide absorption, postabsorption stability and the specific peptides responsible for the effects on REE were neither identified nor discussed. Moreover, the labelling methodology referred to (de Barros et al., 2010; Faintuch et al., 2008) makes use of the introduction of a bifunctional chelating agent to the peptides, to obtain a stable complex (at least 6 h), which is resistant to proteolytic and peptide degradation. The latter is not comparable to the normal situation in vivo. The human protease degradome in vivo comprises more than 500 proteases (Puente et al., 2003) and absorbed food peptides will be substrates for many of these. Indeed, many studies have shown that longer endogenous and exogenous peptides are rapidly degraded in blood by proteases in the plasma and the vascular epithelium and have fast clearance and loss of bioactivity. As discussed above, endogenously secreted satiety and metabolism regulatory peptides all have very short half‐life times (2–6 min; Table 2). For this reason, nonoral routes of peptide delivery, such as infusion and regular injection (Ferreira et al., 2017; Mansi et al., 2021; Pierre et al., 2015) are required to maintain sufficient concentrations and bioactivity. To overcome this, the pharmaceutical industry has worked intensively on strategies for producing molecularly modified peptides, which have a lower susceptibility to degradation (Adessi & Soto, 2002; Muheem et al., 2016).

We therefore conclude that data obtained when using stable radio‐labelled peptides and nonoral routes of delivery are useful to obtain mechanistic insights but cannot be generalised to peptides resulting from the digestion and absorption of food consumed orally. Human data showing sufficient uptake of specific protease‐resistant peptides (of known sequences) and blood levels over time, and a plausible mechanism of action, are required to confirm experimental animal data and allow extrapolation of the data to humans.

## WHAT DO OBSERVATIONAL STUDIES IN HUMANS TELL US?

**FIGURE 3:**
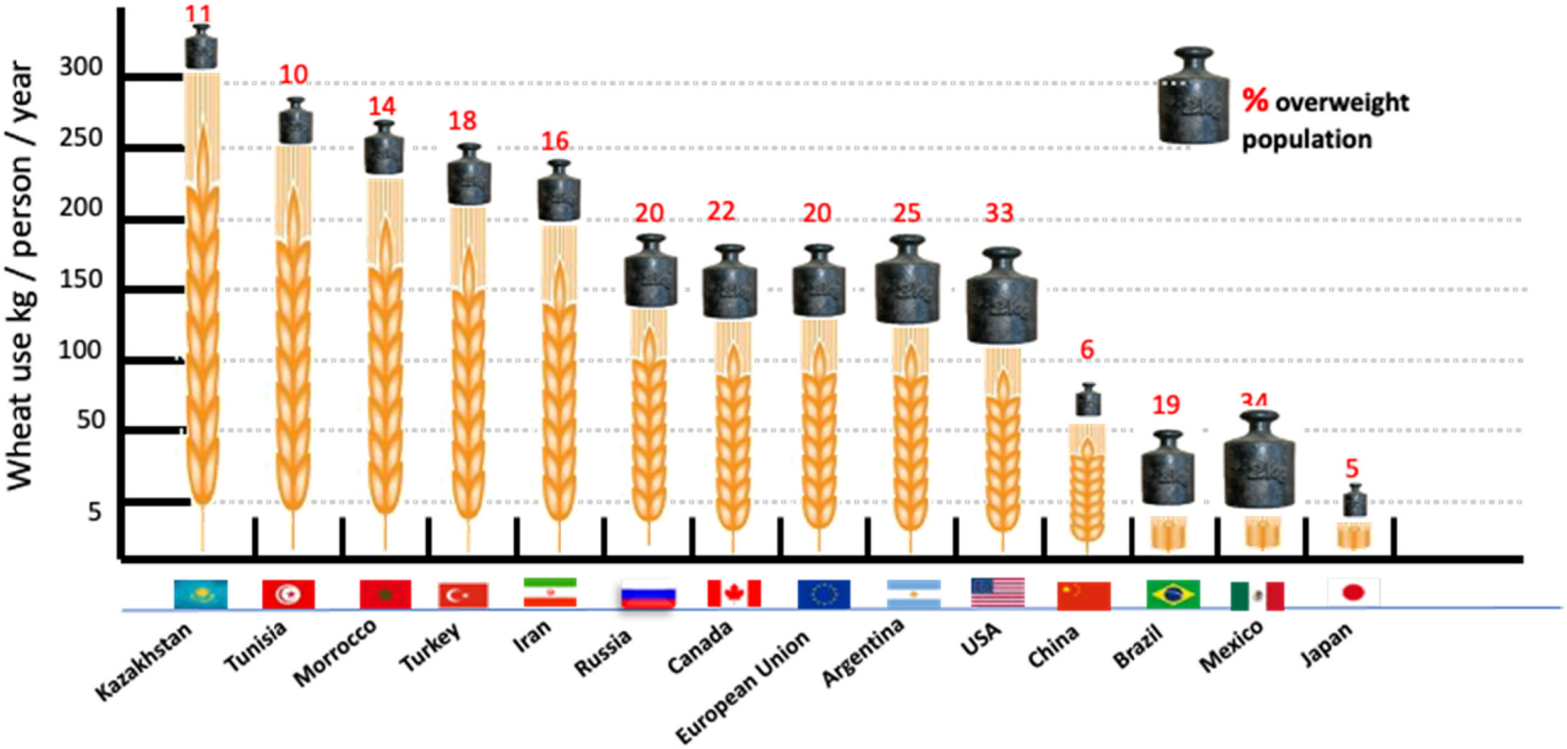
No relation between per capita wheat consumption and obesity estimations based on data from OMS, USDA, OCDE and Canimot. With permission modified from (Braun, 2016)

If the consumption of wheat gluten causes overweight, one would expect a much higher prevalence of overweight in countries where the per capita consumption of wheat and gluten is higher (e.g. North Africa, West and Central Asia), compared with countries where the consumption is low (e.g. Japan, Brazil, Mexico) all else being equal. This appears not to be the case (see Figure 3).

Behrendt et al. (2021) observed a weak positive association between gluten intake and BW in females but a negative association in men in a cross‐sectional study of data from the UK Biobank. The authors referred to this as ‘conflicting findings from the UK Biobank’ and concluded that ‘all observed metabolic health associations related to gluten were weak and not clinically meaningful’. Further, ‘limiting gluten intake is unlikely to provide metabolic health benefits for a population in total’.

No significant effect on BW was observed in a study in which virtually all dietary protein intake was exchanged for wheat gluten as the sole protein source for a duration of 50 days (Bolourchi et al., 1968). Although this study was of short duration and with a limited number of individuals (*n* = 12) it did show that a more than 5‐fold increase in daily wheat protein intake (67 g of wheat protein/day, corresponding to around 500 g of bread and 47 g of gluten/day) did not result in increased BW in any of the subjects. These data strongly indicate that very high levels of gluten intake over 50 days were not associated with an increase in BW in any of the 12 individuals (bearing in mind the small sample size).

**FIGURE 4:**
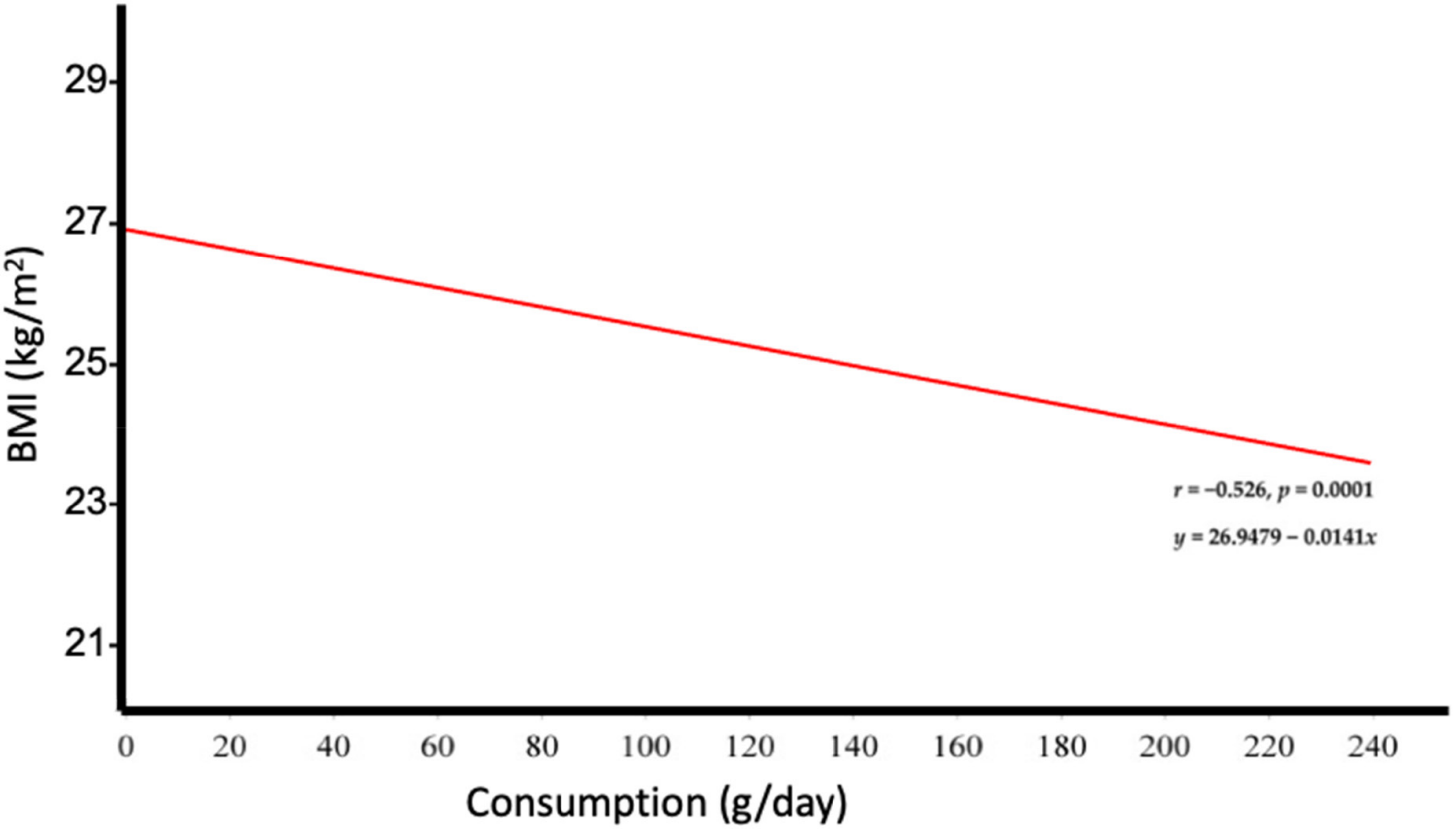
Cross‐sectional and prospective data from observational studies suggest an inverse relationship between whole grain intake and body mass index (BMI). These data are based on a meta‐regression analysis of cross‐sectional data from 12 observational studies (136 834 subjects). With courtesy, modified from Maki et al. (2019)

Maki et al. (2019) reported that observational studies generally show inverse associations between the consumption of whole grain (WG) foods (which usually contain gluten) and weight change within follow‐up periods, which were typically 5 to 20 years (Figure 4).

Similar data were obtained by Schlesinger et al. (2019), who observed a slightly lower risk of weight gain but commented that the certainty of the evidence for less weight gain was rated as low and that further research should provide better evidence. In a more recent meta‐analysis (Sadeghi et al., 2020), the authors failed to find a significant effect of whole grain consumption on any BW parameter. Although the observations listed above are inconsistent in terms of beneficial associations with bodyweight, there is no evidence of a positive association between whole grain foods (containing gluten) and weight gain. Most recently, Wang et al. (2021b) reported data on the long‐term intake of gluten among 13 494 US women, (mean [SD] age, 60.6 [4.6] years), during the years 2014–2019. In this study, there were no differences in total daily calorie intake, or in body mass index between the lowest and the highest gluten intake quintiles. Similarly, data from three other large prospective cohort studies, comprising over 200 000 individuals, showed that total calorie intake and BMI did not differ across the lowest and highest gluten intake quintiles (Wang et al., 2021a).

To help reduce the burden of disease, in particular obesity and chronic diseases, food authorities worldwide recommend regular consumption of whole grain foods (most of which contain gluten), in addition to fruit and vegetables. This is further supported by studies that have shown potentially beneficial effects of specific compounds present in these foods. For example, dietary fibre, micronutrients and phenolic components with antioxidant properties were shown to have anti‐inflammatory properties and favourable effects on gastrointestinal health (Kopf et al., 2018; Seal & Brownlee, 2015).

Available data consistently show that long‐term whole grain consumption is associated with either lower risk of weight gain or no weight gain (Schlesinger et al., 2019; Seal & Brownlee, 2015) and is associated with lower risks of developing type 2 diabetes, cardiovascular disease and intestinal cancers, resulting in general advice to consume whole grains as part of a healthy lifestyle and diet (Aune et al., 2017; Barrett et al., 2019; Gaesser, 2020; Harvard, 2021; Huang et al., 2015; Ley et al., 2014; Lillioja et al., 2013; Maki et al., 2019; Tullio et al., 2020; Wang et al., 2020; Wang et al., 2021a; WHO, 2020; Wu et al., 2015).

## CONCLUDING REMARKS

From the data presented above, it can be concluded that although very small amounts of peptides released from the digestion of gluten and other protein sources may be absorbed, either by transcellular or paracellular transport, most will be rapidly degraded and lose their biological activity. Overall, there is little unequivocal evidence that these peptides, with the possible exception of di‐ and tripeptides, can cross the gut wall and enter the hepatic portal vein and systemic circulation in relevant quantity. This was critically addressed several years ago (Foltz et al., 2010; Miner‐Williams et al., 2014) and the situation has not changed since then. There is still no evidence that peptides are absorbed and are sufficiently stable after absorption, in physiologically relevant quantities to have effects on weight regulation.

Observational studies of gluten intake in very large cohorts show that gluten consumption is not associated with daily energy intake, BW and BMI; findings that have been confirmed in in vivo experimental studies. Indeed, the consumption of whole grain foods, most of which contain significant amounts of gluten, is generally recommended by national and international health authorities to improve the quality of the diet and reduce the incidence of diet‐related chronic diseases.

## CONFLICT OF INTEREST

The authors have no conflicts of interest to declare that are relevant to the content of this article.

## AUTHOR AGREEMENT

All authors have seen and approved the final version of the manuscript.

## Data Availability

Data sharing not applicable ‐ no new data generated, or the article describes entirely theoretical research
